# Design of a multiple criteria decision analysis framework for prioritizing high-impact health technologies in a regional health service

**DOI:** 10.1017/S0266462324000205

**Published:** 2024-04-05

**Authors:** Fernando-Ignacio Sánchez-Martínez, José-María Abellán-Perpiñán, Jorge-Eduardo Martínez-Pérez, Jorge-Luis Gómez-Torres

**Affiliations:** 1 Applied Economics Department, University of Murcia, Murcia, Spain; 2 International Doctorate School, PhD programme in Economics, DEcIDE, University of Murcia, Murcia, Spain

**Keywords:** multiple-criteria decision analysis, healthcare technologies, prioritization, resource allocation

## Abstract

**Objectives:**

This study aims to develop a framework for establishing priorities in the regional health service of Murcia, Spain, to facilitate the creation of a comprehensive multiple criteria decision analysis (MCDA) framework. This framework will aid in decision-making processes related to the assessment, reimbursement, and utilization of high-impact health technologies.

**Method:**

Based on the results of a review of existing frameworks for MCDA of health technologies, a set of criteria was proposed to be used in the context of evaluating high-impact health technologies. Key stakeholders within regional healthcare services, including clinical leaders and management personnel, participated in a focus group (n = 11) to discuss the proposed criteria and select the final fifteen. To elicit the weights of the criteria, two surveys were administered, one to a small sample of healthcare professionals (n = 35) and another to a larger representative sample of the general population (n = 494).

**Results:**

The responses obtained from health professionals in the weighting procedure exhibited greater consistency compared to those provided by the general public. The criteria more highly weighted were “Need for intervention” and “Intervention outcomes.” The weights finally assigned to each item in the multicriteria framework were derived as the equal-weighted sum of the mean weights from the two samples.

**Conclusions:**

A multi-attribute function capable of generating a composite measure (multicriteria) to assess the value of high-impact health interventions has been developed. Furthermore, it is recommended to pilot this procedure in a specific decision context to evaluate the efficacy, feasibility, usefulness, and reliability of the proposed tool.

## Introduction

The growth of healthcare expenditure poses significant challenges to resource allocation in public health systems. Demographic (e.g., aging) and nondemographic determinants of healthcare spending (e.g., biomedical technology innovation) exert considerable pressure on public budgets (1–6). Consequently, healthcare managers face the daunting task of making decisions with substantial opportunity costs within increasingly complex and multifaceted contexts (7;8).

In the European context, a value-based approach is employed to assist in public financing and pricing decisions concerning new health technologies (9). For instance, the United Kingdom assesses value by comparing the cost utility of an intervention (measured as the Incremental Cost per Quality-Adjusted Life Year gained) with an efficiency threshold (10). In France and Germany, however, value is determined based on the incremental therapeutic benefits and domestic reference pricing, with cost-effectiveness playing a small role in the overall approach (11;12).

Furthermore, significant advancements in biomedical innovation have added complexity to the evaluation and decision-making processes (13–15). Due to potential conflicts of interest among stakeholders, there is an increasing use of methodologies that systematize the criteria for assessing health technologies. The multiple criteria decision analysis (MCDA) is particularly notable in this regard, encompassing a set of methods that aid in prioritizing actions by assigning relative importance to each criterion reflecting different dimensions of health technology’s performance. These dimensions include clinical effectiveness, safety, cost, ethical considerations, and patient preferences (16–19).

The aim of this study is to design an MCDA framework to inform decisions on the incorporation of high-impact technologies in the regional health service of Murcia, Spain. By “high-impact” technologies, we mean both impact on patients’ health – reducing the burden of disease they bear, and/or impact on the available budget – consequently displacing other healthcare services.

The Spanish healthcare system is a markedly decentralized one, with a notable degree of autonomy in how each regional health service prioritizes funding for new healthcare technologies, especially those that do not involve pharmaceuticals. Although MCDA is currently used by some Spanish regions (e.g., Catalonia uses this methodology to assess some drugs), in the Region of Murcia – a relatively small Spanish region, accounting for 3 percent of the national population – there is currently no formalized procedure with explicit criteria for making these decisions. This lack of a standardized process results in significant differences between health areas.

The specific objectives are to select the criteria that will be part of the scheme, as well as to obtain the weights of each of them based on the preferences of health professionals and general population. The task of assigning scores to each of the criteria is outside the scope of our study, so in this respect it is similar to the approach followed by Cleemput et al. (20) in their report for the Belgian Health Care Knowledge Centre (KCE).

The next section provides a summary of the fundamental aspects of MCDA and its applications. In Methodology section, we elaborate on the methodologies employed to develop an MCDA framework tailored to assess high-impact health technologies within the context of a Spanish regional health service. The findings derived from the analysis are presented in Results section, followed by a Discussion section, which precedes the final conclusions.

## The multiple criteria decision analysis framework

A classical definition of MCDA is that by Keeny and Raiffa (21), “a methodology for appraising alternatives on individual, often conflicting criteria, and combining them into one overall appraisal.” The potential of MCDA in healthcare decision making was recognized in the 1980s and, since then, the use of MCDA in health technology assessment (HTA) has been actively promoted, based on its potential, but also criticized, because of doubts about its suitability (22). Nevertheless, MCDA has been widely utilized in the healthcare sector for various decision-making purposes (23;24), such as new technology evaluations (25;26), assessment of orphan drugs (27;28), risk–benefit assessments (29), hospital purchasing (30–33), and establishing priority frameworks for different types of interventions (34;35).

Interest in using MCDA to inform decisions on public financing of new technologies has also grown in recent decades. Consequently, various guidelines have been developed based on this methodology by HTA institutions (19;20;25;36).

MCDA is typically categorized into two main approaches: qualitative and quantitative. In qualitative MCDA, technologies are evaluated through deliberation about their performance on explicitly defined criteria (37). The goal of quantitative MCDA is to obtain a global measure of the value of each technology. An overwhelming majority of studies that have utilized MCDA in HTA are of a quantitative nature (37).

Quantitative MCDA frameworks comprise three primary phases (19): selection of criteria, weighting of criteria, and application of the framework established in the two previous phases. The selection of criteria must adhere to the requirements set forth in the recommendation guide of the International Society for Pharmacoeconomics and Outcomes Research (ISPOR): completeness, nonredundancy, no-overlap, and preference independence (18).

Performance for each criterion can be measured using various scales (binary, categorical, ordinal, ratio, interval, etc.). On the other hand, weighting involves eliciting stakeholders’ preferences between criteria (22). Weights reflect the “trade-offs” between criteria and are needed to combine the scores on individual criterion into a unique measure of “total value.”

There are different types of methods for scoring and weighting criteria: direct methods, hierarchical methods, discrete choice methods, and matching methods (38). The source of preferences depends on the type of decision problem. The “stakeholders” can be members of the Regulatory Committees or the Health Technology Assessment Committees, patients, clinical leaders and other health professionals, or the general public (22).

Once the alternatives’ performance is scored and the criteria are weighted, their values must be aggregated to determine which intervention generates the highest value. Aggregation can be performed using a variety of procedures (e.g., additive, multiplicative, regression), depending on the methods used to score the criteria and assign weights (39).

Subsequently, uncertainty analysis in the ADM framework is conducted similar to economic evaluation studies. Sensitivity analysis should consider all sources of uncertainty (structural, stochastic, parameter, etc.), and can be deterministic or probabilistic (40).

## Methodology

### Selection and structuring of the criteria

To select the criteria that will constitute the MCDA framework, a discussion meeting was conducted with a carefully selected group of organizational members. The group included various high-ranking officials from the regional health service, as well as health area managers and other mid-level executives (more detailed information is available in Table S1 of the Supplementary Material 1). All of them possess decision-making authority regarding the purchase and use of these technologies. The meeting took place on November 26, 2021, at the facilities of the regional health service.

Prior to the meeting, the participants were provided with a list of criteria. These criteria resulted from a two-step preselection process conducted by the research team. Firstly, a set of criteria were selected from the latest version of the EVIDEM framework (41). The EVIDEM (Evidence and Value: Impact in Decision Making) framework consists of a “core model” with thirteen quantifiable criteria, grouped into five domains, supplemented by a contextual tool of six qualitative criteria and one criterion related to the opportunity costs of the intervention. Each generic criterion may encompass specific subcriteria pertinent to distinct therapeutic areas or intervention types.

Fourteen criteria were chosen, comprising the thirteen criteria from the “core model” and the Opportunity Cost Considerations criterion. The reason for selecting most of the criteria from the EVIDEM framework was that these criteria are generic and universally applicable (42).

Additionally, the criteria from the KCE framework were integrated, with appropriate modifications when necessary. The KCE report (35) includes results from a survey of the general population and health decision makers, aimed to assign weights to ten criteria grouped into three categories: therapeutic needs, social needs, and the added value of the new treatment. These criteria were based on a transparent decision framework previously developed by the KCE (43), designed to enhance accountability in the realm of public healthcare benefits reimbursement, a goal closely aligned with the objectives of our proposal. Hence, we chose to integrate some of its criteria in our framework.

The criteria thus selected were then grouped into five domains, and are those shown in Table 1, with the exceptions and qualifications indicated at the foot of the table. The precise definition of domains, criteria, and subcriteria can be found in the glossary (Supplementary Material 2).

**Table 1.** Criteria of the MCDA resulting from the focus group

Domains	Criteria	Subcriteria
Need for intervention	• Disease severity • Affected population • Unmet needs	• Impact on HRQoL • Impact on life expectancy — • In effectiveness • In HRQoL • In safety • In convenience
Outcomes of the intervention	• Comparative effectiveness • Comparative safety • Comparative patient–reported outcomes • Type of Benefit^a^	• Change in life expectancy • Change in intermediate results • Change in prevalence — • Change in HRQoL • Change in convenience • Preventive benefit • Therapeutic benefit
Knowledge about the intervention	• Quality of evidence • Expert consensus	• Validity • Relevance —
Economic impact	• Direct healthcare costs • Other healthcare costs • Non–medical costs • Opportunity cost and budget impact	— — — —
Feasibility^b^	• Availability of resources in the system • Organizational impact	— —

Source*:* Own elaboration, based on EVIDEM 10th edition (55), the KCE framework. (35), and the results of the decision makers’ discussion group.

a The criterion “Type of benefit” was initially included as a domain in the proposal submitted for debate and vote. The participants in the focus group agreed to relocate it as a criterion, within the domain “Outcomes of the intervention.”

b The domain “Feasibility” and its two criteria were absent in the initial proposal, but were added as a result of the focus group discussion.

The dynamics of the discussion meeting was as follows: First, the objective and mechanics of the meeting were explained to the participants. The domains were then voted on, followed by a debate and discussion of the results, which, if applicable, could lead to an extension or reduction of the domains. The criteria were then voted on, following the same methodology as for the domains: voting, debate and discussion, and, if necessary, extension, reduction, and/or relocation of the criteria. Finally, this same process was carried out with the subcriteria included within each criterion previously selected.

It is important to emphasize that, before each vote, participants could suggest additions or modifications to the list of domains or criteria under consideration. The objective was to reach final decisions by consensus after discussing the results following each vote. If consensus was not achieved, the majority rule was applied.

### Weighting of the criteria

To obtain the weights associated with the criteria, we conducted surveys with two distinct samples: decision makers and healthcare professionals from the Regional Health Service, and a sample drawn from the general population of the Region of Murcia. This approach allows us to compare the judgments of professionals, who possess specialized expertise, against the presumably less informed viewpoint of the general population.

Sixty-seven professionals were extended invitations by the Regional Health Service to participate in the survey. Among the recipients were area managers, hospital medical directors, coordinators, and heads of specialized services with high technological requirements (surgery, oncology, etc.). The response rate was 52 percent (thirty-five respondents).

A representative sample of the population (n = 500) was obtained through a two-stage stratified sampling methodology. To optimize the response rate, recruitment strategies included advance contact, reminders, and appointment scheduling. As the survey was endorsed by the Health Department, high collaboration was achieved, obtaining a response rate of 99 percent (494 valid questionnaires). Statistics of this sample are available in Supplementary Material 1 (Table S2).

Two questionnaires were designed, and interfaces were programmed for this purpose, with one questionnaire tailored for each sample. The structure of each questionnaire was similar in both surveys, except for the need to include additional information in the case of the general population. In the latter, wording was slightly simplified to ensure comprehension. Both questionnaires started with an introduction to the survey’s primary objective, namely, to determine the relative importance assigned by the respondents to the different criteria within the analysis framework.

The questionnaire for professionals was administered online, while computer-assisted personal interviews (CAPI) were conducted in the homes of participants for the general population sample,.

To assign weights to the domains, criteria, and subcriteria, we utilize the allocation of 100 points. This method involves distributing 100 points among the domains, 100 points among the criteria within each domain, and 100 points among the subcriteria within each criterion. Some screenshots can be seen in Supplementary Material 3.

The weights obtained from the two subsamples were compared by means of parametric (t-test for independent samples) and nonparametric (Mann–Whitney–Wilcoxon) tests.

## Results

### Selection of the criteria

The initial proposal described in the previous section was presented to the eleven members of the discussion group responsible for selecting the criteria. Before voting on the domains, one of the participants suggested adding a domain that captured the availability of resources within the healthcare system to incorporate the technology under evaluation, as well as its impact on the system’s organization. This proposal was accepted by consensus, and the “Feasibility” domain was added, including two criteria (see Table 1). All domains received unanimous support from the participants, except for the “Knowledge of the intervention” domain, which recorded two opposing votes.

The criteria received unanimous endorsement from the participants, with few exceptions: “Comparative safety,” “Patient-perceived outcomes,” “Preventive benefit,” “Therapeutic benefit,” and “Non-healthcare costs” received one opposing vote; the “Expert consensus” criterion was supported by eight out of eleven participants. After a brief debate, participants agreed to relocate the domain “Type of benefit” and its corresponding criteria (“Preventive benefit” and “Therapeutic outcome”) as a criterion within the “Outcome of the intervention” domain.

The subcriteria that did not receive 100 percent of the votes from the attendees were “Unmet needs in HRQoL,” “Change in intermediate outcomes,” and “Change in HRQoL” (one opposing vote each), “Change in convenience” (three opposing votes), and “Unmet needs in convenience” (four opposing votes).

It was understood that all criteria and subcriteria were validated by the participants in the meeting, with the clarifications provided. The final criteria are as shown in Table 1.

### Weighting of the criteria

Table 2 presents the mean weights, accompanied by their standard deviation, for all the domains, criteria, and subcriteria, obtained from each sample. In both cases, the same three domains receive the highest weightings. “Need for intervention” occupies the top position, with a weight of 28.1 percent in the general population subsample and 23.7 percent in the healthcare professionals’ sample. The domain “Intervention outcomes” is ranked second (24.6 and 23.1 percent, respectively), and the third domain is “Knowledge about the intervention” (19.0 and 19.5 percent). In the general population subsample, the fourth-ranking domain is “Feasibility” (14.5 percent). Conversely, healthcare professionals place the domain “Impact on the economy” in fourth position (18.5 percent).

**Table 2.** Weights of the domains, criteria and subcriteria from the two subsamples

	General population		Healthcare professionals	Difference (GP – HCP)		
	Mean	St.Dev.	Mean	St.Dev.	Mean	*P-value*
**I. Need for intervention**	28,08	16,28	23,69	7,60	4,39	0,114
Disease severity	41,10	18,52	36,31	11,55	4,79	0,132
Impact on HRQoL	55,53	20,46	61,31	11,56	−5,79	0,099
Impact on life expectancy	44,47	20,46	38,69	11,56	5,79	0,099
Affected population	31,18	15,88	36,71	9,96	–5,53	0,043*
Unmet needs	27,71	16,86	26,97	9,01	0,74	0,797
In effectiveness	28,16	15,69	31,29	9,51	−3,12	0,246
In CVRS	28,73	14,66	25,40	6,46	3,33	0,183
In safety	22,76	12,06	24,00	6,04	−1,24	0,546
In convenience	20,35	13,30	19,31	5,21	1,03	0,648
**II. Outcomes of the intervention**	24,56	15,89	23,14	6,31	1,42	0,601
Comparative effectiveness	26,59	14,14	28,40	8,30	−1,81	0,455
Change in life expectancy	39,48	19,50	37,74	8,87	1,73	0,602
Change in intermediate results	31,10	17,24	29,54	7,39	1,55	0,597
Change in prevalence	29,43	17,08	32,71	10,00	−3,29	0,262
Comparative safety	23,97	12,96	23,63	6,23	0,34	0,877
Comparative patient reported outcomes	25,31	15,25	24,31	6,83	1,00	0,702
Change in HRQoL	59,18	19,59	61,97	9,88	−2,80	0,403
Change in convenience	40,82	19,59	38,03	9,88	2,80	0,403
Type of benefit	24,13	15,13	23,66	7,15	0,47	0,855
Preventive benefit	52,38	19,89	54,69	11,67	−2,31	0,498
Therapeutic benefit	47,62	19,89	45,31	11,67	2,31	0,498
**III. Knowledge about the intervention**	18,98	12,58	19,46	6,82	−0,48	0,825
Quality of the evidence	59,03	21,23	61,57	10,34	−2,54	0,483
Validity	54,89	18,64	51,34	11,33	3,55	0,267
Relevance	45,11	18,64	48,66	11,33	−3,55	0,267
Expert consensus	40,97	21,23	38,43	10,34	2,54	0,483
**IV. Economic impact**	13,92	9,98	18,54	7,35	−4,62	0,007**
Direct healthcare costs	27,99	14,31	31,86	10,37	−3,87	0,117
Other healthcare costs	25,24	13,58	21,17	5,98	4,07	0,9
Non–medical costs	24,47	14,84	18,51	6,36	5,96	0,019*
Opportunity cost and budgetary impact	22,30	14,56	28,46	11,94	−6,16	0,015*
**V. Feasibility**	14,46	10,43	15,17	5,19	−0,71	0,687
Availability of resources in the system	53,87	20,21	58,66	13,58	−4,79	0,169
Organizational impact	46,13	20,21	41,34	13,58	4,79	0,169

*Source:* Own elaboration. p-values corresponding to the t-test.

The average weight assigned by the general population is higher than that given by healthcare professionals for the first two domains, and lower for the remaining three domains. However, statistically significant differences in mean weights between the two subsamples are observed only in the domain “Economic impact” (p = 0.007).

Regarding the criteria, nine of them receive higher weights from the general population than from healthcare professionals, while six receive lower weights. Nevertheless, statistically significant differences (at the 95 percent confidence level) between the means of the two groups are found only in one criterion of the first domain (“Affected population”) and in two criteria of domain IV (“Non-medical costs” and “Opportunity costs and budget impact”). Lastly, none of the fifteen subcriteria exhibit significantly different weights between the means of the two subsamples.^1^

The analysis of the distribution of absolute frequencies from the combined sample set (N = 529) suggests a greater dispersion of scores in the first two domains compared to the rest, particularly the last two domains. The medians of the scores decrease as one progresses through the domains. The median for the “Need for intervention” domain is 25, followed by 20 for the “Outcomes of the intervention” and “Knowledge of the intervention” domains, and finally 10 for the “Economic impact” and “Feasibility” domains.

**Figure 1.**
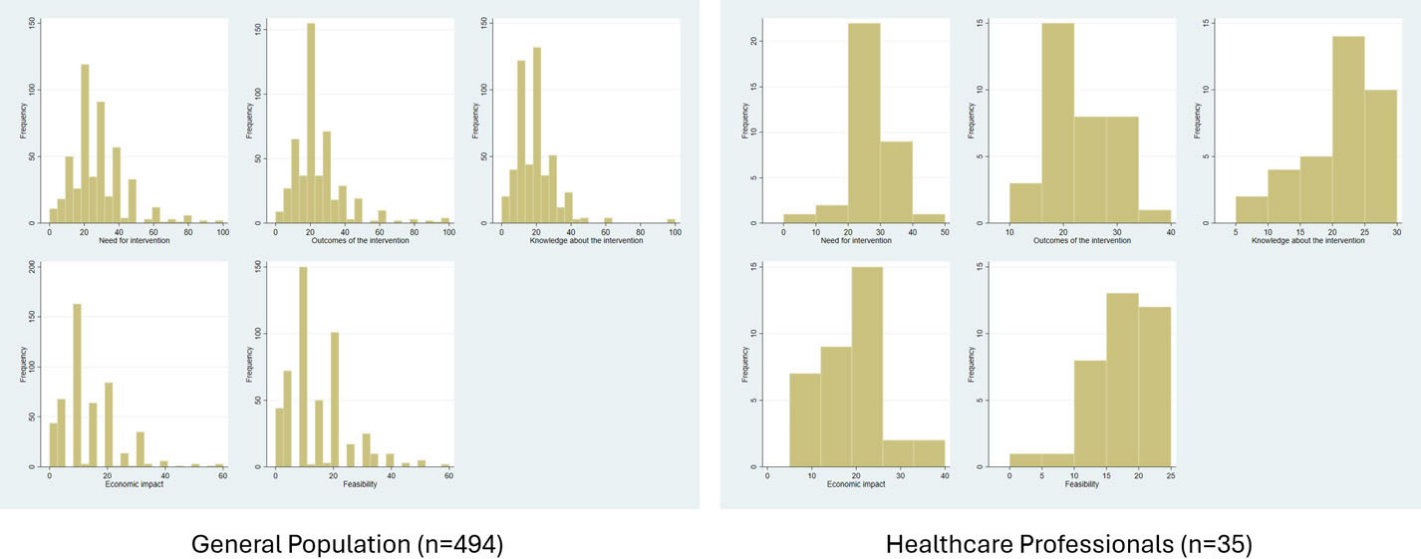
Histograms of the domains’ weights from each subsample.

Differentiating between the two samples, histograms in Figure 1 confirm the higher concentration of weights assigned by the sample of health professionals within a narrower range, typically not exceeding 30, compared to the general population sample, which exhibits a more skewed distribution spreading to the right.

**Figure 2.**
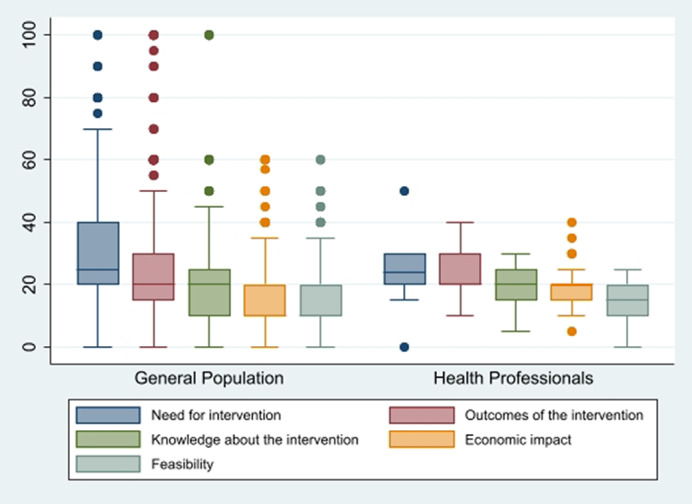
Weights assigned to the domains by each subsample.

In Figure 2, it is evident that the dispersion is significantly higher in the general population sample, although the medians, with the exception of the “Economic Impact” domain and, to a lesser extent, “Feasibility” are very similar. This greater homogeneity of the responses from the health professionals sample extends broadly when comparing the scores assigned to the criteria and subcriteria, as shown in Table 2.

The different nature of the preferences and the significant difference in sample sizes between the two surveys make it impractical to integrate them into a single population to derive a measure of central tendency for establishing the weights. Combining the two samples would inevitably introduce bias toward social preferences, as they represent more than ninety-three percent of the total respondents. Therefore, we propose taking the average of the means obtained in the two samples for each item, that is, an equal-weighted sum of the mean weights from each subsample. By doing so, the resulting weights offer a more appropriate synthesis of both perspectives. These weights, rounded to the nearest integer, are presented in Table 3.

**Table 3.** Weights (%) of domains, criteria, and subcriteria for the MCDA

Domain	%	Criterion	%	Subcriterion	%
Need for intervention	26	Disease severity	39	Impact on HRQoL	58
				Impact on life expectancy	42
		Affected population	34	—	
		Unmet needs	27	In effectiveness	30
				In HRQoL	27
				In safety	23
				In convenience	20
Outcomes of the intervention	24	Comparative effectiveness	27	Change in life expectancy	39
				Change in intermediate results	30
				Change in prevalence	31
		Comparative safety	27	—	
		Comparative patient–reported outcomes	24	Change in HRQoL	61
				Change in convenience	39
		Type of Benefit	24	Preventive benefit	54
				Therapeutic benefit	46
Knowledge about the intervention	19	Quality of evidence	60	Validity	53
				Relevance	47
		Expert consensus	40	—	
Economic impact	16	Direct healthcare costs	30	—	
		Other healthcare costs	23	—	
		Non–medical costs	22	—	
		Opportunity cost and budget impact	25	—	
Feasibility	15	Availability of resources in the system	56	—	
		Organizational impact	44	—	

*Source:* Own elaboration. The weights have been calculated as the average of the means of the two subsamples.

Once the high-impact technology has been valued, by assigning a score to each of the criteria and subcriteria, which falls outside the scope of this article, these scores should be combined with the weights in Table 3 as follows:

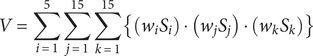

In the formula, *i*, *j*, and *k* represent the domains, criteria, and subcriteria of the analysis framework, respectively. The weightings from Table 3 are denoted as 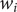 , 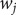 , and 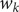 , representing the weights normalized to a total of one. 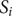 , 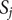 , and 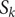 represent the scores assigned by the decision makers to each domain, criterion, and subcriterion of the respective technology being evaluated.

## Discussion

This article develops an MCDA framework for the evaluation of high-impact health technologies in a Spanish Regional Health Service. A multi-attribute function has been developed capable of generating a composite measure to assess the benefits and costs of high-impact health interventions, based on the preferences of healthcare professionals and the general population.

Out of the five domains, “Need for intervention” and “Outcomes of the intervention” are the most highly weighted by both samples. “Affected population,” “Disease severity,” and “Quality of the evidence” ranked at the top among the 15 criteria, a result which is in line with other studies (44–46). While it is true that the first two mentioned domains absorb a 50 percent of the total value of the weighting function, the results also suggest that participants exhibit a certain tendency to distribute points equally between criteria and between subcriteria. This pattern resembles, in some respect, the so-called equalizing bias (i.e., the tendency of decision makers to assign the same weight to different attributes), which seems to affect particularly in point allocation rules, though the bias is less acute under a hierarchical structuring of the decision problem, such as the format used in our study (47). There seems to be also a tendency to use round numbers, which is common in this type of point allocation exercise (48).

Although a remarkable coincidence exists between the weights from the general population and those from the decision makers, some differences arise. First, healthcare professionals give more importance to the economic aspects of the intervention, which coincides with the results of previous studies (49;50). Professionals assigned a weight to the domain “Economic impact” that is 4.5 points higher than the weight assigned by the general population. One possible explanation is that professionals are more aware of the budget constraint and, consequently, more sensitive to the costs of interventions and their economic impact in general.

Another interesting finding is that the dispersion of the weights of the domains is significantly higher in the general population sample than among the decision makers, although the medians turned to be very similar, with the exception of the “Economic Impact.” This greater homogeneity of the responses provided by healthcare professionals seems a logical result, given that, firstly, the shared characteristics among members of this sample (employment status, level of education), as well as the presumably narrower age range it contains, make it more homogeneous. Secondly, it can be assumed that professionals may have more solidly formed opinions, and are therefore less prone to variability. Added to this is the disparate sample size of both groups of respondents, which may also help explain the differences in the degree of dispersion of the responses.

A controversial methodological issue has to do with the inclusion of cost-related attributes among the criteria. There are theoretical arguments for and against (18). It has been argued that the aim of MCDA is to create a composite score of benefit, being the main question to be answered how much money should be spent for one unit of that composite score (51). Some researchers considered as unrealistic to assume that individuals can derive value functions for all criteria including costs and provide weights for the value function of costs in relation to that of the other criteria (37). Regarding cost-effectiveness, specifically, it has been recommended not to include it, from a technical perspective, since it is already a composite of costs and benefits (17). One could assume, even, that the cost-effectiveness criterion, in some way, is implicitly included within the “intervention outcomes” domain (52).

On the other hand, advocates of including costs argue that, by doing so, respondents explicitly make trade-offs between costs and the rest of the criteria, making explicit their contribution throughout the entire decision-making process (53). In a review of MCDA studies to support health technology assessment (37), eighty percent of the studies included costs, and fifty-seven percent included cost-effectiveness, as criteria in the value measurement model. Another systematic review of criteria and scoring functions (54) found that cost-related criteria were considered in more than fifty percent of the selected studies. In our study, we opted for including cost-related criteria in the MCDA framework, as it is the case in some recent studies (49;55).

Incorporating the perspectives of various stakeholders is a fundamental aspect of MCDA. Stakeholder engagement ensures that the evaluation process reflects the values, concerns, and preferences of patients, healthcare professionals, payers, and policy makers, thereby fostering transparency, legitimacy, and acceptance of the final decision. Our study, as the Belgian framework (20), and in contrast to most examples in literature, incorporates the general population in the weighting stage, which is in line with the purpose of the MCDA scheme that has been designed, that is, the incorporation of high-impact technologies into the public system. We think this is one of the strengths of the study, although we acknowledge as a potential limitation of the design the omission of incorporating the perspective of the general population (or the patients’ perspective) in the initial phase of criterion identification.

Despite its advantages, MCDA faces certain challenges and limitations, and our study is no stranger to these. The selection and weighting of criteria can be subjective, leading to potential biases in decision outcomes, and this could be somehow present in our results. Particularly, the method chosen for weighting the criteria, namely, the 100-points allocation procedure, has been regarded as a more prone to framing bias, as criteria and their performance ranges are not explicitly traded off (37). Nevertheless, when choosing a method for weighting, time and resources required, as well as cognitive burden imposed to participants should also be considered (56). The method we chose has the advantage of its simplicity and understandability, and it has been successfully used in previous studies (57).

On the other hand, the advisability of incorporating a deliberative component into any quantitative MCDA has been suggested (37), allowing the decision-making body to carry out a flexible interpretation of the results. This is the spirit that guides the proposal, not that of providing a rigid framework where the score obtained with the multi-attribute function becomes the sole input to consider in the decision-making process.

Finally, validation of the proposed framework would require its application in order to detect possible shortcomings or dysfunctions that could become apparent at the time of its use for the evaluation of a specific intervention or technology. The availability and reliability of data for all criteria could pose practical difficulties. And furthermore, interpreting and communicating the results of MCDA to diverse stakeholders can be complex, demanding effective communication strategies.

Future research, afterwards the framework has been used for a time, could check whether it has indeed been useful for decision makers of the regional health service. A reassessment of its suitability should be done periodically and, depending on its success for making better decisions, to transfer to other instances.

## Conclusions

Multi-Criteria Decision Analysis constitutes a valuable approach to systematically and transparently support decision making, enabling a comprehensive evaluation of healthcare technologies based on various criteria. This article presents a multicriteria decision scheme to guide the purchasing decisions of new high-impact technologies in a Spanish regional health service where, currently, no formal procedure with objective criteria exists for adopting such decisions. The development of the scheme has considered, in its different phases, the preferences of managers, healthcare professionals, and the general population. Although the contributions of the former have shown a higher degree of consistency and lower dispersion than the preferences of the general population, no significant discrepancies have been detected in how criteria are prioritized between the two groups. The result is a multi-attribute function capable of generating a composite measure to assess the costs and benefits of high-impact interventions, with “need for intervention” and “outcomes of the intervention” emerging as the most relevant domains or attributes. Implementing this framework in a specific decision context would provide valuable information about the effectiveness of this tool in informing priority setting in resource allocation within the regional health system.

## Supporting information

Sánchez-Martínez et al. supplementary material 1 Sánchez-Martínez et al. supplementary material

Sánchez-Martínez et al. supplementary material 2 Sánchez-Martínez et al. supplementary material

Sánchez-Martínez et al. supplementary material 3 Sánchez-Martínez et al. supplementary material

## Footnotes

1 When the Mann–Whitney–Wilcoxon test is employed, the statistically significant differences extend to the subcriteria Impact on HRQoL (p = 0.034), Impact on life expectancy (p = 0.034), Change in prevalence (p = 0.043), and Unmet needs in effectiveness (p = 0.043).

## Supplementary material

The supplementary material for this article can be found at http://doi.org/10.1017/S0266462324000205.
