# Supplementary material for: Design of a multiple criteria decision analysis framework for prioritizing high-impact health technologies in a regional health service
Source: Int J Technol Assess Health Care. 2024 Apr 5;40(1):e21. doi: 10.1017/S0266462324000205 (PMC11569904; doi:10.1017/S0266462324000205)
Supplement: Sánchez-Martínez et al. supplementary material 1 — Sánchez-Martínez et al. supplementary material [file S0266462324000205sup001.docx]

**Table S1. COMPOSITION OF THE DISCUSSION GROUP RESPONSIBLE FOR SELECTING DOMAINS AND CRITERIA**

|  | Gender (M/W) |
| --- | --- |
| Director Manager of the Regional Health Service (‘Servicio Murciano de Salud’: SMS) | M |
| Director General of Healthcare of the SMS | W |
| Deputy Director of Healthcare Quality, Safety, and Evaluation of the SMS | M |
| Deputy General Director of Economic Affairs of the SMS | M |
| Deputy General Director of Projects and Innovation of the SMS | M |
| Director General of Hospital Care of the SMS | W |
| Head of Service of the Health Service (Management of Care Coordination Programs) | W |
| Director Manager of Health Area 1 and Virgen de la Arrixaca Hospital | M |
| Director Manager of Health Area 2 and Santa Lucía Hospital | M |
| Director Manager of Health Area 6 and Morales Meseguer Hospital | M |
| Head of the Comprehensive Supply Unit (Procurement Center) of the SMS | M |

**Table S2. SOCIODEMOGRAPHIC CHARACTERISTICS OF THE GENERAL POPULATION SAMPLE**

| **Age and sex** | | | | | | | | | |
| --- | --- | --- | --- | --- | --- | --- | --- | --- | --- |
|  | **Men** | | **Women** | | **Total** | | **Region of Murcia ^(1)^** | |  |
|  | **N** | **%** | **N** | **%** | **N** | **%** | **%** | |  |
| 18 – 29 years | 49 | 19.9 | 45 | 18.1 | 94 | 19.0 | 17.2 | |  |
| 30 – 44 years | 69 | 28.0 | 67 | 27.0 | 136 | 27.5 | 26.6 | |  |
| 45 – 60 years | 65 | 26.4 | 64 | 25.8 | 129 | 26.1 | 30.2 | |  |
| > 60 years | 63 | 25.6 | 72 | 29.0 | 135 | 27.3 | 26.0 | |  |
| Total | 246 | 100.0 | 248 | 100.0 | 494 | 100.0 | 100.0 | |  |
| Average Age (desv.st) | 47 (16.9) | | | | | | | 48.6 | |
| % by sex | 49.8 | | 50.2 | | 100.0 | | 49.8 / 50.2 | |  |
| **Educational level** | | | | | | | | |  |
|  | **N** | | **%** | | **Accumulated Percentage** | | **Region of Murcia (%)** | |  |
| Without studies | 20 | | 4.1 | | 4.1 | | 8.8 | |  |
| Primary | 93 | | 18.8 | | 22.9 | | 8.3 | |  |
| Secondary | 219 | | 44.3 | | 67.2 | | 57.6 | |  |
| Superior | 162 | | 32.8 | | 100.0 | | 25.3 | |  |
| **Civil status** | | | | | | | | |  |
|  | **N** | | **%** | | **Accumulated Percentage** | | **Region of Murcia (%)** | |  |
| Single | 177 | | 35.8 | | 35.8 | | 46.9 | |  |
| Married | 228 | | 46.2 | | 82.0 | | 42.0 | |  |
| Divorced | 57 | | 11.5 | | 93.5 | | 5.9 | |  |
| Widow/er | 32 | | 6.5 | | 100.0 | | 5.3 | |  |
| **Occupation** | | | | | | | | |  |
|  | **N** | | **%** | | **Accumulated Percentage** | | **Region of Murcia (%)** | |  |
| House husband/wife | 29 | | 5.9 | | 5.9 | | 10.9 | |  |
| Student | 41 | | 8.3 | | 14.2 | | 9.9 | |  |
| Retired | 92 | | 18.6 | | 32.8 | | 15.1 | |  |
| Own account | 68 | | 13.7 | | 46.6 | | 9.4 | |  |
| Employed | 201 | | 40.7 | | 87.3 | | 46.2 | |  |
| Unemployed | 63 | | 12.8 | | 100.0 | | 8.5 | |  |

1. Spanish National Institute of Statistics: Census and Labor Force Survey. 2022.
