# Supplementary material for: Design of a multiple criteria decision analysis framework for prioritizing high-impact health technologies in a regional health service
Source: Int J Technol Assess Health Care. 2024 Apr 5;40(1):e21. doi: 10.1017/S0266462324000205 (PMC11569904; doi:10.1017/S0266462324000205)
Supplement: Sánchez-Martínez et al. supplementary material 2 — Sánchez-Martínez et al. supplementary material [file S0266462324000205sup002.docx]

**GLOSSARY OF DOMAINS, CRITERIA AND SUBCRITERIA**

**Disease severity**: severity of the health condition of the patients treated with the intervention (or severity of the condition that is intended to be prevented) in relation to mortality, morbidity, disability, functioning, impact on quality of life, disease course (intensity, clinical stages). The impact of disease severity on health-related quality of life (Impact on HRQoL), as perceived by patients, as well as on survival (Impact on life expectancy) is considered.

**Affected population**: number of people affected by the condition (treated or prevented with the intervention) among a specific population in a given period of time. It can be expressed as the annual number of new cases (incidence) and/or as the proportion of the population affected at a specific moment in time (prevalence).

**Unmet needs**: deficiencies of the interventions being compared in their capacity to prevent, cure or mitigate the health problem to which they are addressed (Effectiveness). Included among these deficiencies are those corresponding to safety (Safety), the results perceived by patients (HRQoL) and convenience or comfort (Convenience).

**Comparative effectiveness**: capacity of the intervention to prevent or produce a desired (beneficial) change in the symptoms or in the course of the condition superior to the beneficial changes generated by alternative interventions. This change is captured in the form of increased life expectancy (Life expectancy), better intermediate or surrogate results (Intermediate results) and prevalence (Prevalence).

**Comparative safety**: ability of the intervention to produce a reduction in unwanted or harmful effects related to the intervention compared to those caused by alternative interventions (Adverse events).

**Patient-reported outcomes**: ability of the intervention to produce beneficial changes in patient-perceived outcomes greater than the beneficial changes produced by alternative interventions. It includes changes perceived by patients in terms of health-related quality of life (HRQL) as well as convenience or ease of use (Convenience).

**Type of benefit**: if the intervention yields a preventive benefit or risk reduction (eg eradication, prevention, reduction in transmission, reduction in the prevalence of risk factors) or a therapeutic benefit (eg relief of symptoms, prolongation of survival, cure).

**Quality of the evidence**: extent to which the available evidence on the intervention is relevant (*Relevance*) for decision-making (in terms of population, disease progression, comparators, outcomes, etc.) and valid (*Validity*) with respect to scientific standards (study design) and previous conclusions (degree of concordance of the results obtained between different studies). Includes considerations about the uncertainty of the evidence (e.g. conflicting results between studies or a limited number of studies and patients). Having a complete report of the available evidence is a prerequisite to assess its coherence and validity.

**Expert consensus**: degree of suitability of the intervention (or of similar alternatives) according to the existing consensus among experts about what constitutes appropriate medical practices for the management of the health condition in question. The recommendations of the experts in this regard are usually made explicit in the form of clinical practice guidelines with the intention of improving the quality of this.

**Direct healthcare costs**: net cost of financing the intervention, understood as the difference between the expected cost of the intervention and that corresponding to other interventions that could be replaced by it. This comparison is limited to the cost directly linked to the intervention, that is, the cost of acquisition, implementation and maintenance.

**Other health costs**: impact of the intervention on other health costs (excluding the direct cost of the intervention), such as hospitalization costs, specialized consultations, those caused by possible adverse effects, long-term care, etc.

**Non-medical costs**: impact of the intervention on non-health costs, such as those corresponding to social services, productivity losses, informal care, etc.

**Opportunity cost and budget impact**: consideration of the health resources that may be sacrificed (opportunity cost) as a result of the implementation of the intervention and the system's capacity to assimilate such intervention budgetarily.

**Availability of resources in the system**: material and, above all, human resources available to the system to guarantee a priori the implementation of the intervention, without the need to displace other services or programs in force. The displacement, where appropriate, of these would be included in the preceding criterion (“Opportunity cost and budgetary impact”).

**Organizational impact**: magnitude of the reorganization of available resources that must be undertaken, if applicable, in order to address the considered intervention.
