# Supplementary material for: Design of a multiple criteria decision analysis framework for prioritizing high-impact health technologies in a regional health service
Source: Int J Technol Assess Health Care. 2024 Apr 5;40(1):e21. doi: 10.1017/S0266462324000205 (PMC11569904; doi:10.1017/S0266462324000205)
Supplement: Sánchez-Martínez et al. supplementary material 3 — Sánchez-Martínez et al. supplementary material [file S0266462324000205sup003.docx]

**SOME EXAMPLES OF THE WEIGHTING PROCEDURE**


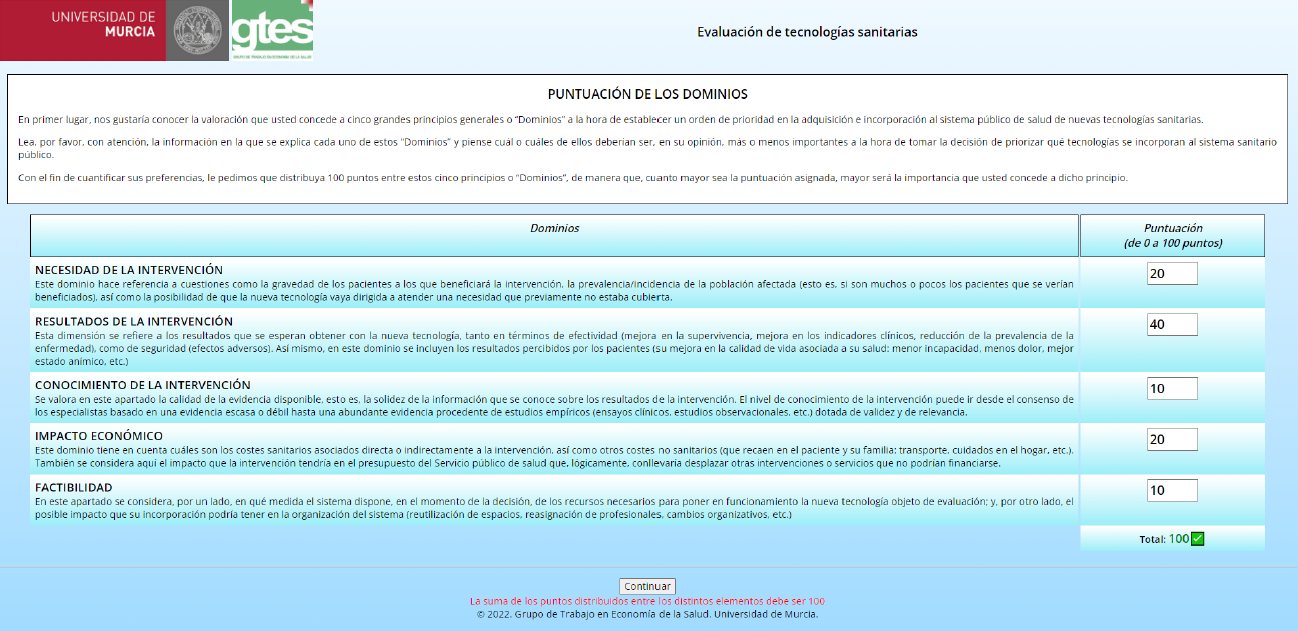


*Figure 1. Example of distribution of the 100 points among the 5 domains*


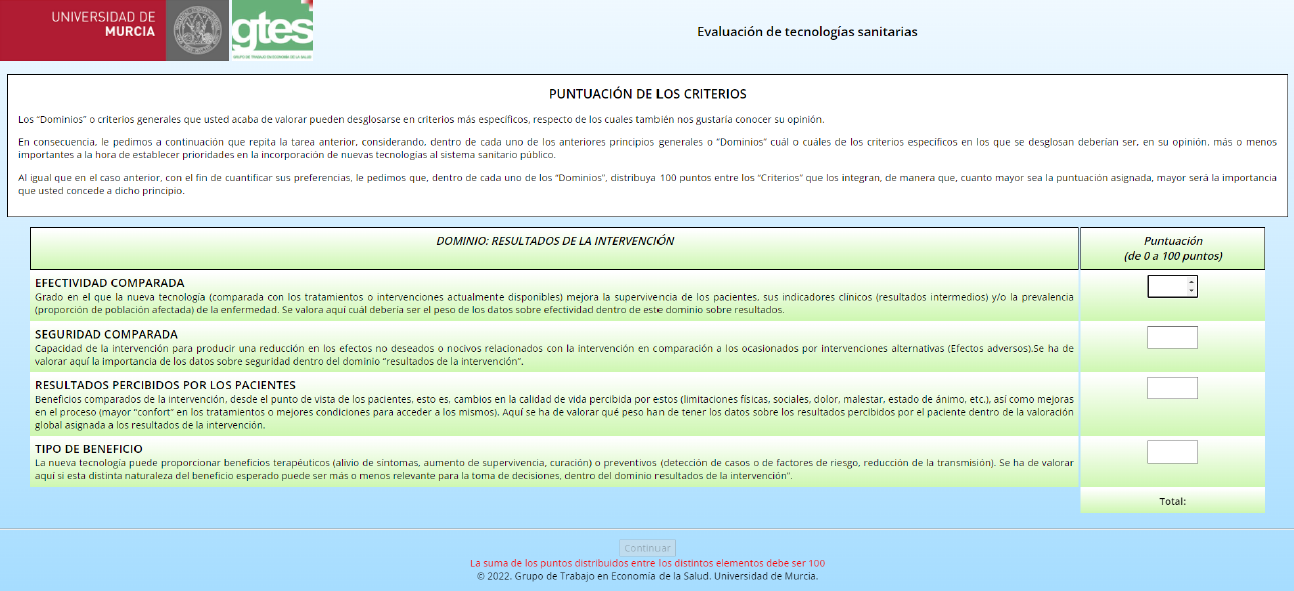


*Figure 2. Weighting task of the criteria of the “Results of the intervention” domain.*
